# Challenges of Future Patient Recruitment: A Cross-Sectional Study in Conservative Dentistry Teaching

**DOI:** 10.3390/dj13110495

**Published:** 2025-10-25

**Authors:** Marco M. Herz, Michael Scharl, Diana Wolff, Valentin Bartha

**Affiliations:** 1 Department of Conservative Dentistry, Tuebingen University, Osianderstr. 2-8, 72076 Tübingen, Germany; 2 Private Practice, Wolfgang-Brumme-Allee 25, 71034 Böblingen, Germany; michael.scharl@student.uni-tuebingen.de; 3 Department for Conservative Dentistry, Heidelberg University, Im Neuenheimer Feld 400, 69120 Heidelberg, Germany; diana.wolff@med.uni-heidelberg.de (D.W.); valentin.bartha@med.uni-heidelberg.de (V.B.)

**Keywords:** dental school, dental patients, dental education, dental care economics

## Abstract

**Background:** Direct clinical training on real patients is essential in dental education. However, the declining patient inflow increasingly challenges this objective. This cross-sectional study aimed to assess patients’ experiences and preferences to derive recommendations for improving patient recruitment. **Material and Methods:** Over a period of one year, patients treated by students in the courses and final examinations at the dental school of conservative dentistry were questioned using a specially designed questionnaire and reviewed using their medical records. They were asked about their complete treatment process, and patient files were used to record socio-demographic as well as economic and appointment-specific data. **Results:** We analysed 297 patients (142 women, 47.8%; 155 men, 52.2%) treated by undergraduates across two semesters (four courses) and two final examinations. Median age was 57.0 years (IQR 46–67; mean 55.2, SD 15.2; range 14–85) with no sex-based difference (*p* > 0.05). Arrival was predominantly by car (72.7%, n = 216); median one-way distance was 20.5 km (IQR 11.2–32.1); and 58.4% were employed, while 41.6% were not employed (33.7% retired, 7.9% unemployed). The leading reason for course attendance was “satisfaction with previous treatments” (65.32%). Information sources were reported by 290/297 (98%); the most common was already being a course patient (143, 48.1%). Most patients attended one appointment (109, 36.7%). Median travel cost per appointment (including parking) was €17.0 (typically €10.0–€23.5). Of 285 respondents, 93.68% answered “Yes” to satisfaction with student treatment. **Conclusions:** Important steps include enhancing parking facilities, optimizing recall systems and appointment accessibility, and strengthening relationships with regular patients to encourage word-of-mouth referrals. The main focus is to maintain high clinical quality, ensure affordability, and further reduce patient copayments where possible.

## 1. Introduction

Worldwide, many patients continue to seek treatment from dental students for a variety of reasons. Ahmady et al. (2015) identified five key dimensions in a literature review that shape patient satisfaction with dental school treatment: quality, interaction, access, environment, and cost [1]. Butters and Willis (2000) similarly found that, across different patient groups, quality of care, the length and frequency of appointments, clarity of treatment explanations, and the level of fees were essential determinants of satisfaction [2].

The most important reason cited in many studies remains the cost advantage: treatments at university dental clinics are often significantly less expensive or even free. This makes dental care more accessible and attractive, particularly for low-income populations or patients with limited access to regular dental services [3,4,5,6]. However, student-provided treatment often requires more time, as preliminary assessments and multiple supervisory steps lead to longer treatment durations and waiting times compared to private practices [7]. Economically, this corresponds to a higher “opportunity cost”, which some patients perceive as a disadvantage [7].

Nevertheless, many patients benefit from the high availability of appointments in university clinics. In some regions, this easy access has even shortened waiting lists, since the student care model provides additional treatment capacity [8]. Overall accessibility is also high: university clinics often accept young children and elderly patients without complex admission procedures [5,8].

Beyond financial relief, the perceived quality of care is a central factor. Dental students tend to work with particular care and thoroughness, as each step is supervised by experienced faculty members, resulting in structured and closely monitored treatments perceived as high quality [9,10,11]. In Germany, this is institutionalized through the “six-eyes principle,” which ensures continuous quality control by involving at least two supervising professionals for each treatment step, which further enhances therapeutic safety [12].

The fact that students take ample time and respond individually to their patients is widely perceived as a positive aspect [13]. University dental clinics also often offer shorter waiting times and faster appointment scheduling than many regular practices, which is particularly advantageous in urgent cases [6]. This high level of patient satisfaction is also linked to the close supervision provided by faculty members: every student’s treatment is continuously monitored, and each step of the procedure is carefully reviewed, resulting in greater thoroughness and quality in care delivery [7,14]. Students are also often perceived as particularly empathetic and communicative [15,16]. Altruism itself is a relevant factor: many patients consciously choose student clinics to contribute to the education of future dentists [6,14].

Despite many positive experiences, recurring points of criticism are also addressed in the studies. Foremost among these is the time factor: due to the students’ limited routine and the extensive supervisory mechanisms, appointments usually take longer than in a regular practice. While, according to Grimme, 76% of patients were satisfied with the waiting time for follow-up appointments, only 65% expressed satisfaction with the on-site waiting time [17]. Nevertheless, many patients accept the longer treatment durations when they are accompanied by high-quality care. Even though the treatment time is generally longer—for example, in prosthetic treatments, which according to Huettig and Behrend (2016) require approximately twice as many appointments—many patients are willing to invest this additional time because they benefit from close supervision and cost savings [18]. Another study even showed that patients subjectively experienced less strain during treatment in the student clinic compared to regular practices—primarily because the students took more time for individualized care. [12]. In addition, a change of student during an ongoing treatment could disrupt the trust relationship or affect the dentist–patient relationship [19]. Organizational factors such as travel to the clinic, appointment scheduling, or the clarity of procedures also play a role in shaping patient satisfaction [11].

However, patient numbers at university clinics are declining. Despite clear advantages, fewer people opt for student care. This trend raises concerns about public perception, clinic organization, and competition from low-cost private providers—issues that deserve more attention to secure the model’s future.

Strategies or concepts are therefore necessary to counteract this trend—for example, by addressing patient needs and motives more effectively through modifications in procedural workflows or by placing greater emphasis on aspects that are perceived positively. Studies offering concrete strategies for improving patient retention beyond analyzing patient motives remain limited.

The scarcity of such studies was, therefore, one of the key motivations for designing the present study. The core idea and guiding research question were: “Can patients’ opinions, preferences, and potential criticisms serve as a basis for developing recommendations or strategies to improve patient recruitment and retention?”

This question was to be addressed through a questionnaire, aiming to systematically capture and analyze both the contextual factors and the decision-making processes of course patients regarding treatment by dental students, as well as to comprehensively examine the entire process from appointment scheduling to treatment. The results are intended to provide a deeper understanding of the patient population and thus identify potential approaches to enhance patient recruitment in university dental clinics. No confirmatory null hypothesis was formulated because the study was descriptive and exploratory in nature.

## 2. Materials and Methods

This cross-sectional observational study descriptively analyzed participants treated by dental students in the patient courses and final examinations of the Department of Conservative Dentistry at the Dental School of the University of Tübingen between November 2020 and October 2021. Data were collected using a purpose-designed questionnaire and supplemented with information extracted from patient records. Participation was voluntary and required written informed consent from all patients.

This study was designed as a cross-sectional study and approved by the local ethics committee (number 120/2020BO2/02, 20 April 2020) and registered at the German Clinical Trials Register (DRKS) under ID DRKS00029310, 16 January 2023. All participants gave written consent and could withdraw from the study at any time.

All participants gave written informed consent and could withdraw from the study at any time.

### 2.1. Recruiting Patients

In principle, the Dental Clinic of the University Hospital Tübingen, Germany, operates as a walk-in facility that is open to all patients seeking dental care. The reasons for attending the clinic vary widely. The following entries, documented in patients’ medical records, illustrate the main motives for presenting at the central admissions department, either for the first time or again after an extended period:

○ Attendance based on recommendations from acquaintances, family members, or colleagues
○ Seeking treatment at a lower cost compared with private dental practices
○ Expectation of up-to-date, evidence-based care and corresponding treatment options
○ Absence of a regular family or general dentist
○ Proximity of the clinic to the patient’s residence or workplace

Perception that treatment in a university clinical environment is more objective, or a wish to obtain a second opinion

As the dental clinic has limited control over the number of incoming patients, the student courses depend on the availability of individuals who

1. Attend the dental clinic in general, and
2. Request treatment within the student courses.

Before a patient is accepted, specific eligibility criteria are evaluated by a supervising dentist from the Department of Conservative Dentistry to determine whether treatment within the department’s student courses is both feasible and consistent with the patient’s preferences.

### 2.2. Inclusion Criteria

- Provision of informed consent for treatment by dental students
- Therapeutic complexity appropriate to the students’ level of education and clinical competence

Exclusion criteria:

- Presence of infectious diseases, severe systemic illnesses, or potentially life-threatening conditions (e.g., HIV, Hepatitis C, immunosuppression, leukaemia, dementia, or high-risk anticoagulation status)
- Lack of reliability or compliance as a course participant

If a patient chooses to participate in the student course, they are informed in advance about the treatment setting and procedure, the anticipated treatment measures, the typically greater number of visits (often twice as many as in private practice), the longer appointment durations, and the potential cost savings.

The patient is then registered with the central administration of the student courses in the Department of Conservative Dentistry and scheduled for the next available appointment.

**Figure 1:**
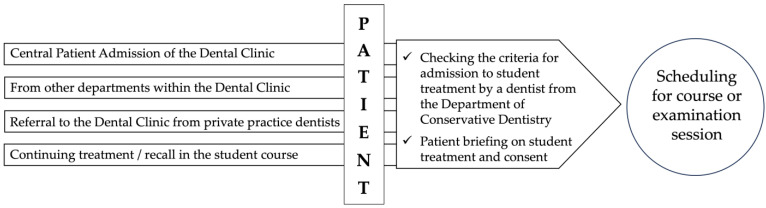
Patient Admission Process for Student-Delivered Treatment in Conservative Dentistry.

Figure 1 illustrates the referral pathway to the central course administration of the Department of Conservative Dentistry and the subsequent progression through the courses leading to the final examination.

### 2.3. Treatment Settings and Procedures

After an initial screening, patients are allocated according to case complexity: advanced cases are assigned to higher-semester students, and simpler cases to earlier semesters. The courses are conducted in year 4 (Course I) and year 5 (Course II) each comprising morning and afternoon sessions. The state examination takes place within five months after completion of all courses, which together include 14 treatment hours distributed over five days.

Patients may be transferred between courses for specific procedures.

Except in cases of acute pain, each course begins with a comprehensive assessment and professional cleaning. Based on the findings, students—together with a supervising dentist—develop a prioritized treatment plan, and patients are informed about procedures and associated costs. Both courses cover conservative dentistry (prophylaxis, direct restorations, periodontal therapy, root canal treatment), while Course II additionally includes partial ceramic crowns, endodontic re-treatments, and paediatric care. Each course enrolls up to 31 students who alternate between the roles of practitioner and assistant across 12 dental chairs; during each half-day session, one dentist supervises four chairs (eight students).

**Figure 2:**
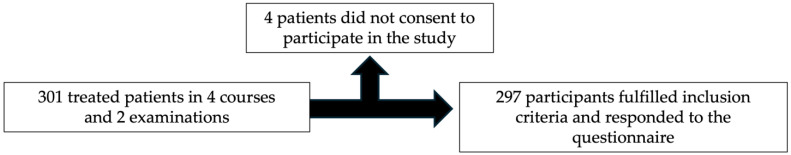
Flowchart of participant selection.

The survey was conducted over one year, from November 2020 to October 2021, encompassing two semesters and two final examinations: the winter semester 2020/2021 (W20), the summer semester 2021 (S21), and examinations 1/21 (E1) and 2/21 (E2). During this period, 301 patients were treated, of whom 4 declined or withdrew from study participation without providing reasons. Figure 2 presents a flowchart of participant selection.

### 2.4. Procedure of the Survey and Questionnaire Design

**Figure 3:**
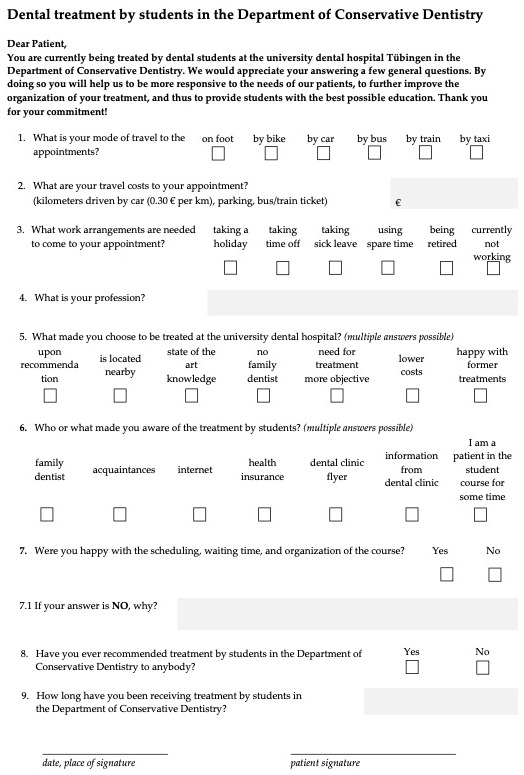
Translated questionnaire for dental treatment by undergraduate students.

The paper questionnaire covered the dental-school appointment (Figure 3). After development of the questionnaire, it underwent internal review by two faculty members for clarity and content before distribution. Items offered predefined responses, allowing multiple selections where specified. Options were drawn from comments in patient records, using the most frequently noted remarks.

Additional questions addressed course organisation, scheduling, and willingness to recommend. At the first course appointment, patients received brief study information at the Department of Conservative Dentistry’s registration desk plus a consent form and questionnaire. They could read these in the waiting area, consider participation, and, if willing, complete the questionnaire before treatment. Remaining questions or uncertainties could be discussed with the treating students.

A pilot survey involving only a subset of patients, as well as the planned qualitative interviews, could not be conducted due to the COVID-19 pandemic.

### 2.5. Calculation of Travel Distance and Travel Costs

Travel distance to the clinic was determined using Google Maps from the patient’s locality (not the exact address). We used the mean of the shortest and fastest routes; if this were identical, an alternative available route was used; if only one route was available, it was selected. The transport mode indicated in the questionnaire was applied: car, taxi, bus, or train were assigned the car route; while bicycle and walking used the respective route. Per-appointment travel costs were calculated as round-trip distance × €0.30/km (the 2020 statutory allowance) for car, bus, or train; none were applied for bicycle or foot. Total travel costs were obtained by summing all attended appointments. Car users paid an additional €5 parking fee per appointment, with no additional charges for other modes. In addition to this objective cost calculation, patients were also able to indicate their travel expenses in the questionnaire themselves (e.g., €0.30 per kilometre of travel, parking fees, etc.), which enabled a secondary calculation referred to as self-reported travel expenses.

### 2.6. Calculation of Treatment Appointments and Treatment Duration

All student appointments were documented. A session was defined as any morning or afternoon period during which both team members were present, regardless of procedures. Session duration was derived from recorded start and end times; incomplete records were excluded. Total treatment time represented the sum of rounded session durations; mean session length was calculated by dividing total time by the number of documented appointments. The first treatment date was obtained from the patient record; the diagnosis was extracted from chart entries and, when available, treatment plans. Waiting time was defined as the number of days between diagnosis and treatment start, applied equally to both new and returning patients. From the records, we can determine the treatment year within the student’s course and whether care was extended beyond two years. The first session in the analysis period served as the reference point, and entries from the prior two years were included. Questionnaire responses were used to validate the documentation.

### 2.7. Calculation of Patient Age

Patient age at the time of study participation was calculated as the difference between the date of birth and the date of study consent.

### 2.8. Calculation of Treatment Costs, Co-Payments, and Savings

To assess patients’ overall costs, we summed all treatment costs, co-payments, and savings across all appointments in the observation period, drawing on medical records, stored co-payment forms, and documented travel routes. Records contained information on the number of appointments, procedures, and fixed statutory insurance benefits (according to BEMA). Additional patient-borne costs were obtained from co-payment forms; these were billed in accordance with GOZ/GOÄ (Fee Schedule for Dentists or Physicians), could be multiplied by a complexity-based adjustment factor, and were subject to departmental discounts. Table 1 illustrates two typical billing examples from conservative dentistry as encountered in the student course.

Discounts/Reduction: Patients treated by students receive a ‘compensation for effort’, that is, a reduction in total costs (dental fees plus material/laboratory) after subtracting contributions from other payers. During the observation period, treatments within the student course were discounted by approximately 40–50%, while certain state-exam treatments received a full (100%) discount. Euro amounts were taken directly from patient invoices, and each patient’s total discount was documented accordingly. To assess the overall financial burden, we added patients’ travel and parking costs. Actual savings equalled were calculated as the granted discount minus these additional expenses. All calculations were expressed in euros.

### 2.9. Sample Size

Since no predefined primary hypothesis was formulated, we did not conduct an a priori sample size calculation. Instead, given the observational nature of this study, we applied a time-based census approach and included all participants who met the inclusion criteria and attended the course within a predefined two-semester period. Accordingly, the sample size was determined by case availability rather than by statistical power considerations. Results are therefore interpreted in an exploratory manner, and effect estimates are reported together with 95% confidence intervals.

### 2.10. Handling of Missing Data

Item nonresponse was coded as missing and excluded from the denominators of the respective analyses. Unless stated otherwise, percentages refer to valid responses per item; totals may therefore not always sum to 100%. No imputation of missing data was applied.

### 2.11. Statistical Methods

Data were collected using a custom-built input form built on the Ninox database platform (Ninox v3.7.14; Ninox Software GmbH, Berlin, Germany) and subsequently exported to Microsoft Excel (v16.69.1; Microsoft Corporation, Redmond, WA, USA). Statistical analyses were conducted using JMP 16 and 18.2.1 (SAS Institute GmbH, Heidelberg, Germany). Differences were considered statistically significant at *p* < 0.05 and highly significant at *p* < 0.001. Descriptive statistics comprised absolute and relative frequencies. For continuous variables, means and standard deviations were calculated; for non-normally distributed data (Anderson–Darling test, *p* < 0.05), medians and interquartile ranges were reported. For between-group comparisons involving non-normally distributed continuous variables, the Mann–Whitney U test (two-sided α = 0.05). Associations between categorical variables were assessed using Pearson’s χ^2^ test of independence. Binary (logistic) regression was performed to evaluate the effects of predictors on the binary dependent variable. Categorical predictors were dummy-coded relative to a reference category and continuous predictors were entered as linear terms. Given the descriptive cross-sectional design, these inferential tests were exploratory and intended to identify potential associations rather than to confirm predefined hypotheses. Consequently, findings should be interpreted as hypothesis-generating.

## 3. Results

The study included a total of 297 participants, 142 women (47.8%) and 155 men (52.2%), who were treated over two semesters within four courses and two final examinations in the Department of Conservative Dentistry by undergraduate students. The median patient age was 57.0 years, with half of the patients aged between 46 and 67 years old at the time of treatment. The mean age was 55.2 years (SD = 15.2), and patients’ ages ranged from 14 years to 85 years. There was no statistically significant difference in the age distribution between male and female patients (*p* > 0.05). An overview of patient characteristics is provided in Table 2.

### 3.1. Travel Distance, Mode, and Costs

**Figure 4:**
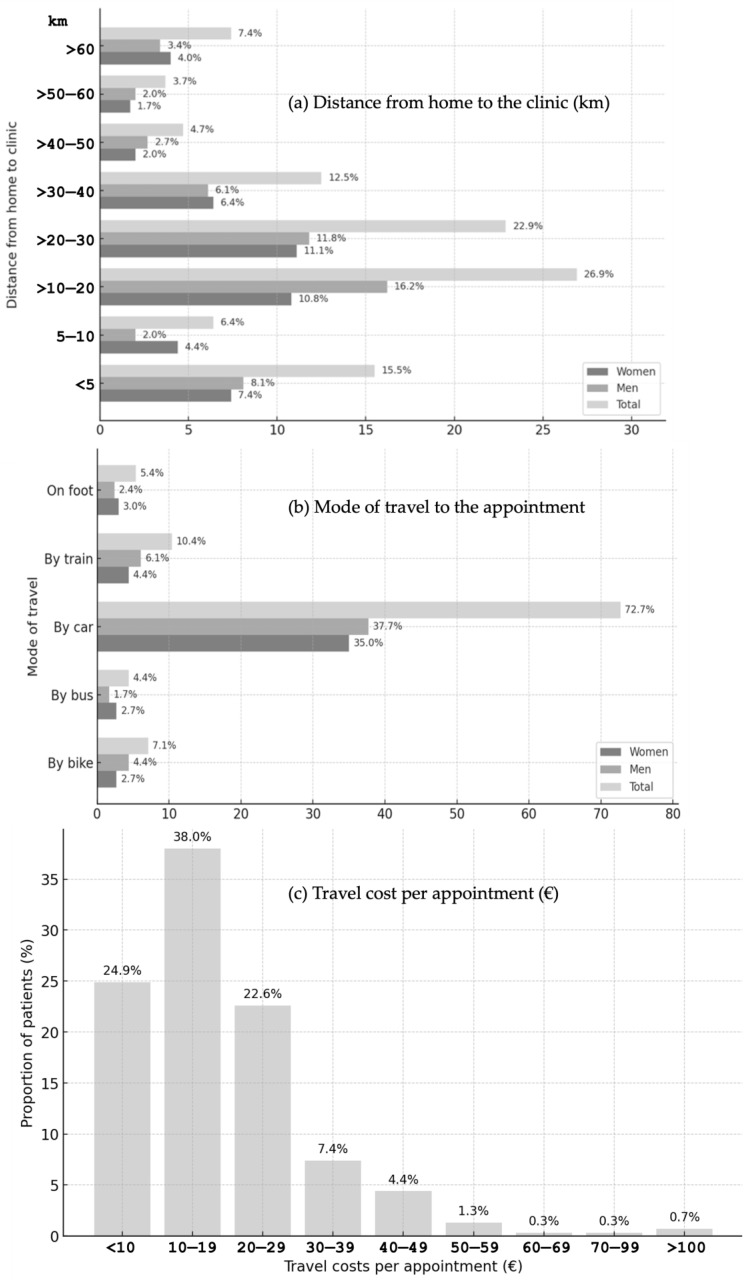
Distance to the clinic (**a**), mode of travel to the appointment (**b**), and travel costs (**c**).

The median one-way distance between participants’ residences and the clinic was 20.5 km (IQR: 11.2–32.1 km) (Figure 4a).

All 297 participants provided information on their mode of transport to the appointment. The most frequently used mode was the car (n = 216; 72.7%), followed by the train (n = 31; 10.4%) and the bicycle (n = 21; 7.1%) (Figure 4b).

Calculated travel costs: Travel was cost-free for 37 participants (12.45%) who walked or cycled, while 260 (87.5%) incurred expenses travelling by car, bus, or train. Thirty-seven participants (12.45%) paid less than €10 per appointment, and the largest group, 113 participants (38.0%), spent €10–19 per appointment (including parking). Higher costs generally corresponded to fewer participants, with a slight increase once expenses exceeded €100 (Figure 4c). The median travel cost per appointment, including parking, was €17.0 (IQR: €10.0–23.5), and reported costs ranged from €0 to €134.1. Across all appointments during the observation period, the median total travel cost was €30.6 (IQR: €14.2–67.4), with a minimum of €0 and a maximum of €663.3 (Figure 4c).

Furthermore, we examined whether employment status was associated with travel distance, but no significant association was found; median distances were comparable between employed and unemployed participants (Mann–Whitney U test, *p* > 0.05).

Self-reported travel expenses: Among 174 respondents, 97 participants (55.7%) reported—based on subjective estimates—travel costs of less than €10 per appointment. Of these, 19 participants (10.9%) travelled on foot or by bicycle and therefore reported no travel costs. As travel costs increased, the proportion of affected participants declined steadily, with the exception of the €50–59 range. The median self-reported travel cost per appointment was €8.0 (mean = €9.3, SD = €8.6). The middle 50% of responses ranged from €4.0 to €12.0 (IQR = €8.0), and reported costs ranged from €0 to €60.0 (range = €60.0).

### 3.2. Work Arrangements for Appointment Attendance

**Figure 5:**
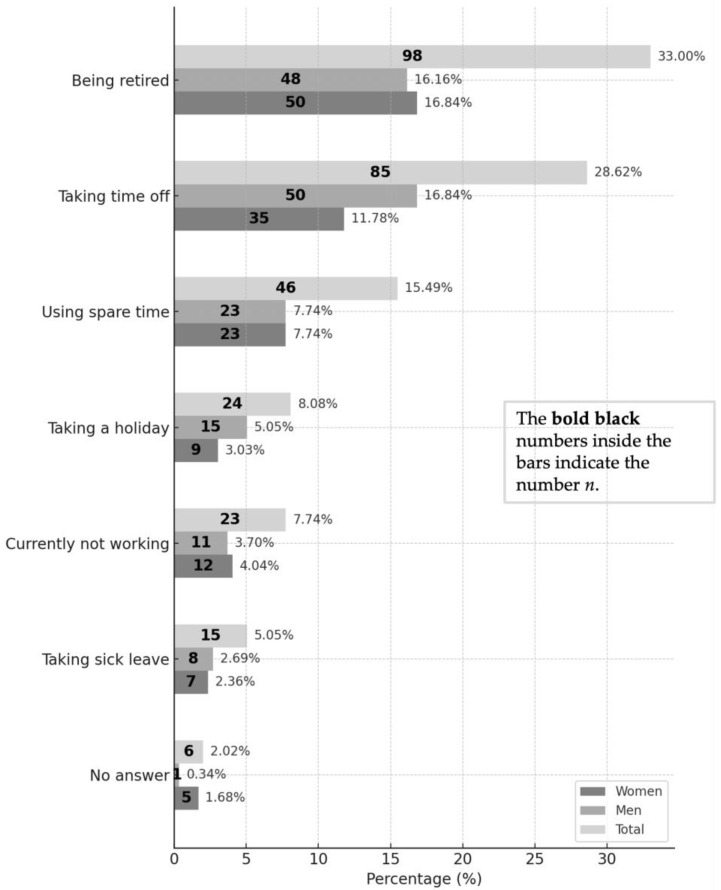
Work arrangements for appointment.

Information on attendance-related work arrangements was available for 291 participants. The most common category was “retired” (n = 98; 33.7%), followed by “took time off work” (n = 85; 29.2%) and “attended during leisure time” (n = 46; 15.8%). The complete frequency distribution is presented in Figure 5.

### 3.3. Reasons for Seeking Treatment at the Dental Clinic

**Figure 6:**
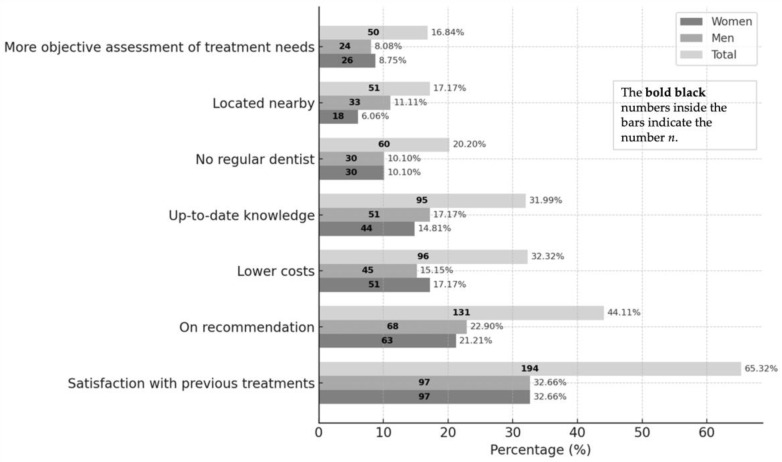
Reasons for seeking treatment at the Tübingen Dental Clinic.

Among 295 respondents (multiple responses permitted), the most frequently cited reason for seeking treatment was satisfaction with previous treatments (65.3%; women and men 32.7% each). Recommendations ranked second (44.1%; men 22.9%, women 21.2%). The largest gender difference was observed for proximity, reported by men 11.1% vs. women 6.1% (Δ = −5.0%), a statistically significant difference (Pearson’s χ^2^ test, *p* = 0.049). Women more often mentioned lower costs (17.2% vs. 15.2%; Δ = +2.0%). The least frequently reported reason was seeking a more objective assessment (16.8%; women 8.8%, men 8.1%). The complete distribution of reported motives is shown in Figure 6.

We examined whether the distribution of stated reasons for seeking the student course differed by sex. Only “proximity” showed a difference: men cited proximity more often than women (21.3% [33/155] vs. 12.7% [18/142]; Pearson’s χ^2^(1) = 3.87, *p* = 0.049).

We also investigated whether the distribution of stated reasons varied by employment status. Among all motives, only “satisfaction with previous treatments” differed significantly: unemployed participants cited this reason more frequently than employed participants (75.4% [52/69] vs. 60.1% [89/148]; Pearson’s χ^2^(1) = 4.80, *p* = 0.029).

### 3.4. Sources of Information About the Student Treatment

Among 290 respondents (multiple responses permitted), the most frequently reported source of information was being or having previously been a course patient (48.15%; women 24.24%, men 23.91%). The next most common sources were acquaintances (42.1%; gender not specified) and clinic information (24.9%; men 13.1% vs. women 11.8%).

Other sources were reported only rarely, including the internet (3.4%), regular dentists (3.4%), clinic leaflets (2.7%), and health insurers (0.3%).

**Figure 7:**
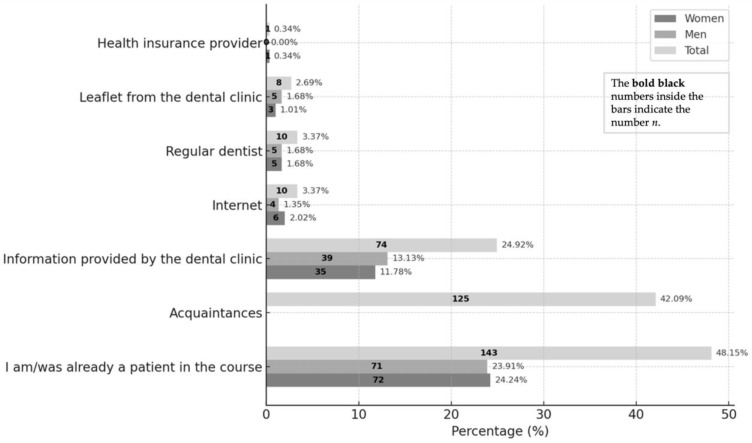
Ways of obtaining information about student treatment.

The largest gender differences were observed in internet use, which was slightly higher among women (+0.7%), and in clinic information, cited more often by men (−1.3%). The complete distribution of information sources is presented in Figure 7.

Only thirteen of the 297 participants (4.4%) were treated in the course following an external referral.

### 3.5. Number and Duration of Treatment Appointments

**Figure 8:**
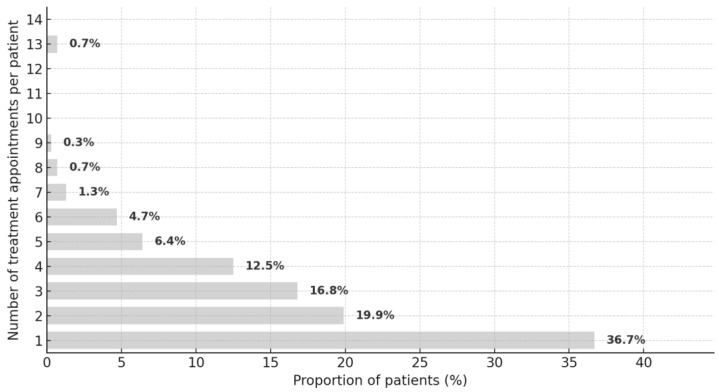
Number of treatment appointments.

Most patients attended only one appointment (n = 109; 36.7%), while 59 (19.9%) had two, 50 (16.8%) had three, and two patients (0.7%) had 13 appointments (Figure 8). The median number of appointments was 2 (mean = 2.6, SD = 1.9; IQR = 1–4), with a minimum of 1 and a maximum of 13 (range = 12).

The frequency of appointments declined as the number of visits increased—from one (n = 109; 36.7%) to nine (n = 1; 0.3%)—with a slight increase at 13 appointments (n = 2; 0.7%). The complete distribution is presented in Figure 8.

From 185 valid responses, the total treatment time had a median of 4.0 h (mean = 6.1 h, SD = 5.2 h). For 50% of participants, the total treatment time ranged from 2.5 to 8.0 h (IQR = 5.5 h). The minimum total treatment time was 0.8 h, and the maximum was 35.3 h (range = 34.5 h).

The average treatment time per session had a median of 2.6 h (mean = 2.6 h, SD = 0.6 h). For 50% of participants, the average treatment time per session ranged from 2.0 to 3.0 h (IQR = 1.0 h). The minimum average session time was 0.8 h, and the maximum was 4.3 h (range = 3.5 h).

### 3.6. Financial Aspects of Student-Provided Dental Treatment

**Figure 9:**
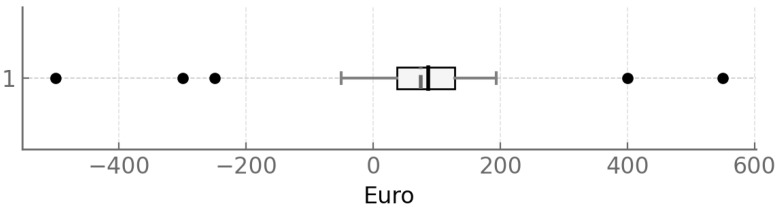
Boxplot of savings after deduction of opportunity costs.

Across all appointments, the median dental fee was €276.4 (IQR = €182.5–€451.8; range = €0–€2015.4). Material and laboratory costs were typically €0.0 (maximum = €508.7). Other contributions (e.g., BEMA) had a median of €47.3 (maximum = €810.3), and the student-treatment discount had a median of €107.6 (IQR = €70.0–€172.7; maximum = €538.7), resulting in a median out-of-pocket expense of €118.1 (IQR = €79.7–€186.0; maximum = €1040.0) and a median net saving—discount minus travel cost—of €71.9 (IQR = €32.7–€110.5; range = −€508.6 to €538.7). A box plot illustrating this distribution is presented in Figure 9.

### 3.7. Satisfaction with Course Organization and Treatment

**Figure 10:**
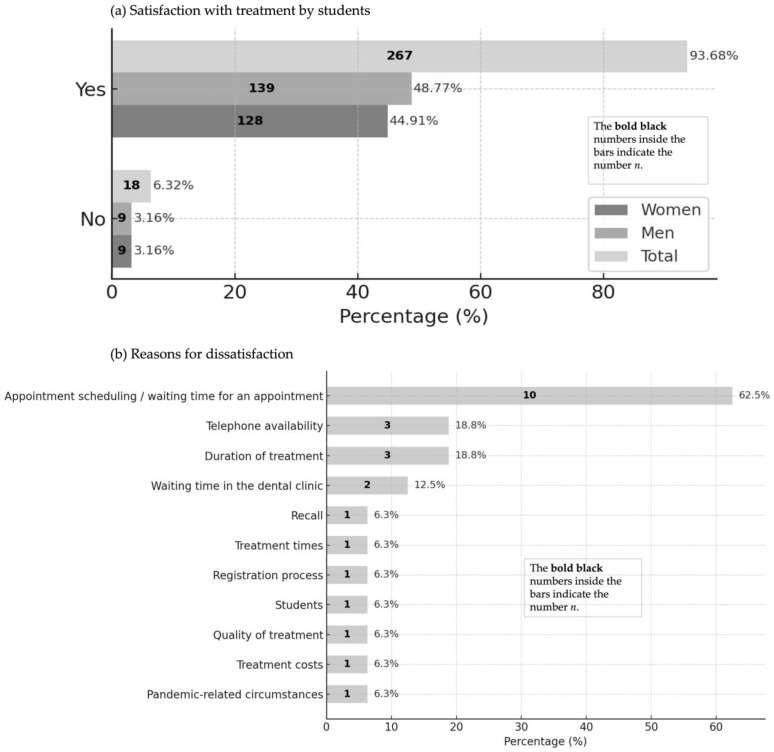
Satisfaction with treatment (**a**) and reasons for dissatisfaction (**b**).

Of the 285 participants, the vast majority responded “Yes” (93.7%), with similar proportions among women (44.9%) and men (48.8%). Only 6.3% responded “No”, equally distributed between women and men (3.2% each). Overall, men accounted for a slightly larger proportion of the sample (51.9%) than women (48.1%) (Figure 10a).

In total, 79.4% of the 287 respondents reported that they had already recommended the student treatment to others. The most frequently cited reasons for dissatisfaction (multiple responses permitted) were scheduling and waiting times (62.5%), followed—at considerably lower frequencies—by telephone availability and treatment duration (each 18.8%) (Figure 10b).

### 3.8. Determinants of Satisfaction

Two factors were associated with satisfaction (Table 3): higher overall self-reported travel costs were linked to lower satisfaction, and patients citing “objective assessment” or “lower costs” reported lower satisfaction. All other tested variables showed no association (Supplementary Table S1).

### 3.9. Previous Time in the Courses

According to the patient records, 126 participants (42.4%) had been treated by students for more than two years, whereas 171 participants (57.6%) had received treatment for less than two years. The duration of previous treatment in the student course showed no significant association with patient satisfaction.

## 4. Discussion

The study included 297 participants treated over two semesters in four courses and two final examinations in the Department of Conservative Dentistry by undergraduate students. Compared to the German average age of 44.6 years (2020) [20], participants were older (mean 55.2 years). Among 295 respondents (multiple answers allowed), the main attendance reason was satisfaction with previous treatment (65.32%), followed by recommendation (44.11%). A gender difference emerged regarding proximity (men 11.11% vs. women 6.06%; *p* = 0.0493), likely reflecting mobility patterns (commuting, car use) rather than perceived quality, consistent with the car-dominant travel profile.

The median one-way distance was 20.5 km; travel modes were mainly car (72.7%), train (10.4%), and bicycle (7.1%). Among 291 participants, attendance was most often during retirement (33.7%), time off work (29.2%), or leisure (15.8%). Information sources (n = 290) were led by former course patients (48.15%), acquaintances (42.09%), and clinic information (24.92%). Most attended once (36.7%). Of 285 respondents, 93.7% were willing to return (women 44.9%, men 48.8%), and 79.4% (n = 287) had recommended the service. Eighteen dissatisfied participants mainly cited scheduling/waiting times (62.5%), telephone access (18.8%), and treatment duration (18.8%). Records showed 42.4% had received treatment for >2 years. Unemployed participants more often emphasized prior positive experiences, likely due to lower opportunity costs and stronger ties to the clinic.

Treatments delivered in dental schools are markedly less expensive and broaden access—especially for lower-income groups—while maintaining quality. Several studies consistently report that patients choose student-provided care primarily due to the reduced or free treatment costs. [1,2,5,9,20]. For instance, in the study by Habib et al., 67% of participants reported that low cost was their main reason for attending the university clinic [9]. In Germany, a dissertation at Witten/Herdecke University reported that 37.3% of respondents cited financial reasons as their primary motive for visiting the student clinic [17]. In Hong Kong, replacing time-based tariffs with fee-for-service reduced patient charges to roughly 20–40% of market prices, increased output by about 15%, doubled cost recovery from 20% (1996) to 40% (1999), preserved or improved satisfaction (DSI 64.5/65.1 pre-reform; 66 among students and 70 among staff/affiliates post-reform), shortened waits from about two months to ≤6 weeks, and shifted non-use from “cost” to “no need/lack of time” [21,22].

Consistent ADEA surveys report fees below usual-and-customary private rates and substantial revenues from student care—$6313 per third-year and $11,680 per fourth-year student in 1998/99 [23,24,25]. Efficiency gains matter: four-handed assistance raised daily patient contacts from 1.74 to 2.62 (+50%), a change that likely reduces waits and opportunity costs [26]. Because total cost to patients equals fees plus time and travel, savings can diminish at higher incomes when time costs dominate, and co-payments shape the out-of-pocket burden [7]. Student-run free clinics generally improve access and satisfaction, though referrals may disrupt continuity [27]. In resource-limited settings, treatment costs and explanations often score lowest, with differences by residence (*p* < 0.0001) and sex (*p* < 0.05) [28].

In the present study, the median travel cost was €17.0 per appointment (range €0–€134.1) and €30.6 over the observation period (range €0–€663.3). Self-reports were lower (€8.0 per appointment), indicating underestimation. The average dental fee was €276.4 (range €0–€2015.4); material/laboratory costs had a median of €0 (range €0–€508.7); and statutory insurer contributions (e.g., BEMA) had a median of €47.3 (range €0–€810.3). Fee reductions yielded a median saving of €107.6 (range €0–€538.7), with out-of-pocket expenses of €118.1 (range €0–€1040). After accounting for travel, the actual saving was €71.9 (range −€508.6 to €538.7). Losses occurred mainly with long, paid travel, whereas the largest gain was observed in a bicycling patient with 13 appointments. Opportunity costs rose with distance; the main beneficiaries were local patients with no travel expenses. Median session length was 2.6 h (range 0.8–4.3 h), and total treatment time was 4 h (range 0.8–35.3 h) across a median of two appointments, reflecting student routines and supervision. Overall, treatment is most advantageous when travel is short and the scope modest; many visits or long distances erode the benefit. These findings argue for outreach to nearby residents, continued efficiency improvements, and clear communication of both monetary and time costs.

Catchment and travel patterns further contextualize access. Although average dental travel in Germany is ~7 km (longer in rural areas), this study observed a 25 km one-way distance—close to the 26 km previously reported for Tübingen prosthodontics [18,29]. Internationally, most attendees lived ≤20 km away in Sri Lanka [30], and 55% were ≤20 km at Peradeniya (28.1% < 10 km; 27.5% 10–20 km), with high satisfaction [31]. In the UK, urgent-care travel averaged about 25 km before falling as additional hubs opened [32]. Of course, such findings cannot be readily generalized to the whole of Germany. A German facial-pain clinic showed a 15–20 km peak, median 39 km, mean 45 km, and maximum 180 km, illustrating supra-regional pull for specialized services [33]. Locally, maxima were about 12–15 km within the city and 30–35 km across the district. Modes skewed toward the car (73%) and train (10%), implying a reliance on parking and a profile distinct from inpatient-oriented specialist clinics.

Despite longer treatment times and care provided by less experienced students, the present study demonstrated exceptionally high patient satisfaction: around 94% of respondents reported being satisfied and approximately 79% had already recommended the student course to others, placing the University of Tübingen slightly ahead of the dental schools in Hamburg and Witten/Herdecke [11,17]. International data support these findings: Lafont (1999) reported that 99%of patients surveyed in English-speaking countries would recommend student treatment, although long treatment and waiting times were the main points of criticism [34], and Butters & Willis (2000) likewise found that recommendations and perceived quality—together with low costs—were key motivators, while criticisms concerned treatment quality, clarity of explanations, number of appointments, and costs [2]. Across settings, patients treated in dental-school clinics report good to very high satisfaction, typically >75–80%, with several studies approaching about 99% [2,9,34,35,36]; concrete estimates include 80% in Saudi Arabia [37], about 76% at the University of the West Indies, with “quality” scoring highest and “access” lowest [35], about 99% in Costa Rica [38], and nearly 99% in the UAE, with pain patients particularly satisfied (*p* < 0.001; [39]). Determinants of a positive experience include empathic attention, professional and technical competence, positive relationships, fulfilled expectations, and clear communication [40].

Beyond these overall levels, patterns in determinants of satisfaction emerged. Higher cumulative travel costs coincided with lower satisfaction, plausibly because monetary and time burdens (including parking and coordination effort) are more salient to these patients and may erode perceived value. In addition, patients attending primarily for “lower costs” or an “objective assessment” tended to be more critical. This is consistent with stronger price salience, higher expectations, and, in the latter case, prior dissatisfaction or diagnostic uncertainty that heightens scrutiny of the encounter.

Awareness channels were dominated by word-of-mouth (45%), with websites contributing 3% and flyers/posters 2%. About a quarter of patients were referred by university clinicians—often for financial constraints or complex care—while 3% came via private dentists and none via statutory insurers. Within current German and UK rules, informative and non-misleading advertising is permitted, whereas superlatives, inducements, and deceptive claims are prohibited, with case law addressing non-neutral listings. Responsibly used advertising and public relations can build volume; social media is effective yet raises professionalism and ethics issues. A multi-channel strategy that pairs outreach with reputation and experience management is advisable, as boundary-crossing claims remain off-limits. Untapped potential lies in local media, collaborations with medical and non-profit providers, and visibility at health events. As continuing-education and master’s pathways now enable private dentists to offer modern, evidence-based care, universities have largely lost exclusivity over “state-of-the-art” therapy—making clarity on value, efficiency, and patient experience all the more important. Syntheses highlight core experience domains—quality/competence, communication, access/organization, environment, and cost/value—and point to practical levers: transparent fee-for-service discounts (20–40%), efficiency measures (four-handed work, appointment management) that can deliver about 50% productivity gains, strengthened patient education with streamlined referral/recall, and explicit management of co-payments and time costs [1].

As outlined in the introduction, there is a notable scarcity of studies that provide concrete strategies for enhancing patient retention and attracting new patients to university dental clinics.

In her dissertation, Frank found that patient satisfaction strongly depends on empathetic, person-centered care; therefore, she emphasizes that social and communication skills must be firmly integrated into dental education [11]. Klaassen et al. identified, among other factors, emotional support, clinical competence, expectation management, and clear communication as key determinants of satisfaction; based on these findings, they recommend targeted training in communication and expectation management to strengthen patient-centered practices [40].

Tashakandi et al. found that patients praised the quality of treatment and professionalism but criticized organizational shortcomings such as waiting times; consequently, they advocate improvements in scheduling while maintaining the existing high standards of clinical and communicative quality [41]. Gutierrez-Marin et al. observed that more frequent visits were associated with higher satisfaction, but also that differences existed among departments and patient groups; therefore, they recommend regular patient surveys and the harmonization of accessibility and quality standards [38].

Fredericks-Younger et al. showed that most complaints were related to management and quality issues, particularly concerning finances, delays, and the overall patient experience; they thus call for structural improvements in organizational management [42]. Finally, Chongkonsatit et al. found that all seven dimensions of the “7Cs Marketing Mix” influenced satisfaction, particularly “Caring” and “Clinic Environment”; accordingly, they recommend targeted investments in empathy, attentiveness, and a high-quality clinical environment [43].

This study reveals three overarching areas derived from the findings that may serve as focal points for concrete recommendations or potential changes, which are summarized below.

Motivation and Patient Experience

Patients’ participation was primarily motivated by satisfaction with previous treatments, complemented by personal recommendations, reduced costs, and confidence in evidence-based care. Recruitment occurred mainly through word-of-mouth, with traditional advertising playing only a minor role. The large proportion of long-term patients indicates stable retention, while key criticisms concerned long waiting times and limited telephone accessibility.

Sociodemographic Characteristics

The mean participant age was 55.2 years—above the national average—with a slight predominance of men. Although most were employed, many were retirees, reflecting an older and socioeconomically less advantaged group. The median one-way distance to the clinic was 20.5 km, with most patients coming from the city and surrounding areas and travelling by car.

Cost–Benefit Assessment

Despite extended waiting times, lengthy treatment sessions, and occasional travel expenses, most patients viewed the approximately 50% reduction in co-payments as a decisive advantage, particularly for complex or repeated treatments. Overall, financial benefits outweighed temporal and logistical burdens, and potential disadvantages due to travel distances were largely accepted.

### 4.1. Limitations of the Study

#### 4.1.1. COVID-19 Pandemic

This study unfortunately took place under extremely challenging conditions, namely during the COVID-19 pandemic. At that time, the duration and full impact of the pandemic were entirely unpredictable. It affected all aspects of daily life and inevitably influenced the study process as well. Fortunately, apart from the mandatory shutdown period, patient treatment in the student courses continued without interruption or substitution by simulation units. Despite these concerns, the study was initiated, as the issue of declining patient numbers had already become apparent before the pandemic, and timely insights into potential solutions were urgently needed. A planned pilot testing with a random patient sample could therefore not be implemented; instead, the final questionnaire was used directly.

Nevertheless, a direct impact of the pandemic on the study cannot be ruled out. During this period, numerous public warnings and behavioral recommendations were issued, particularly to vulnerable groups. Older patients and those with multiple comorbidities were advised to postpone elective appointments and to avoid indoor settings with several people present for extended periods—conditions typical of student course environments. In addition, public transport use was discouraged to minimize infection risk. These factors likely contributed to an underrepresentation of older patients and a lower total number of treated cases compared to non-pandemic semesters. This clearly represents the main limitation of the present study. A future repetition or updated version of this survey would therefore be highly advisable.

#### 4.1.2. Further Limitations

Some of the questionnaire items, particularly those addressing patients’ perspectives, were designed and developed based on documentation in the patient records. Suitable standardized or previously validated items from other studies were not identified in the literature, were deemed not appropriate, and were therefore not applied. This may represent a potential limitation regarding comparability with other studies.

It should also be noted that the results are based on subjective responses from a patient cohort whose behavior and concerns may have been influenced in some way by the concurrent pandemic and its associated circumstances.

The question about occupation did not allow for reliable conclusions regarding income, as the responses were often too imprecise. Instead, they were used to examine employment status: 68.2% of participants were employed (including trainees and volunteers), 31.8% were not. The validity is limited, since, for example, retirees could be classified differently depending on their responses. Additionally, the question about availability for the appointment showed that 58.4% gave responses indicating employment (leave of absence, vacation, etc.), while 41.6% were not employed, of whom 33.7% were retired and 7.9% unemployed.

### 4.2. Conclusions

In light of the circumstances potentially associated with the pandemic and the limitations outlined above, the following conclusions can be drawn:

- The student courses and examinations in conservative dentistry at the University Dental Clinic of Tübingen represent a successful care model for a specific patient population that is older than average and primarily drawn from the local area.
- The parking infrastructure must be adequate and, where necessary, improved; the incurred parking fees could either be explicitly reimbursed or implicitly accounted for within the treatment invoice.
- Established patients constitute a crucial backbone of the course and are likely a major driver of word-of-mouth referrals; therefore, their recall must be systematically maintained.
- Improvement potential: appointment scheduling and accessibility.
- Long-term priorities: maintain high medical quality, promote targeted interdisciplinary training, ensure economic attractiveness for patients, and secure a steady influx of patients to meet the growing demand for complex, patient-centered dental care—adequate public funding remains essential to maintain affordable treatment fees and ensure high-quality clinical education.

## Figures and Tables

**Table 1:** Two cost-related examples of typical treatments in conservative dentistry.

Treatment		By Dentists	By Students
**Professional mechanical plaque removal** (PMPR) of 25 teeth (1 visit)			
1. Fixed dentist’s fee according to health insurance		Free of charge for patients with statutory health insurance	
2. Copayment—Special services not covered by insurance		€405	0.5 × €405 = €202.50
3. Travel costs for 1 visit	travelling	(2 × €0.30 × 20 km) = €12	
	parking	€4	
	total	€16	
4. Treatment discount minus travel costs = **financial benefit**		€421	€186.50
**Direct composite fillings** on one tooth (First upper molar: mesio-occluso-distal filling) (1 visit)			
1. Fixed dentist’s fee according to health insurance		Free of charge for patients with statutory health insurance	
2. Copayment—Special services not covered by insurance		€132.50	0.5 × €132.50 = €66.25
3. Travel costs for two visits	travelling	(4 × €0.30 × 20 km) = €24	
	parking	€8	
	total	€32	
4. Treatment discount minus travel costs = **financial benefit**		€164.50	€34.25

Note: The patient lives 20 km from the clinic (median one-way distance) and travels by car.

**Table 2:** Patient characteristics.

	Total		Women		Men	
	n	%	n	%	n	%
Age Groups						
<20	2	0.67	2	0.67	0	0.00
20–29	25	8.42	9	3.03	16	5.39
30–39	24	8.08	13	4.38	11	3.70
40–49	39	28.62	16	5.39	23	7.74
50–59	82	27.61	43	14.48	39	13.13
60–69	71	23.9	33	11.11	38	12.79
70–79	46	15.49	24	8.08	22	7.41
≥80	8	2.69	2	0.67	6	2.02

**Table 3:** Univariable associations between candidate predictors and satisfaction (Yes vs. No): χ^2^ tests for categorical variables and logistic regression for continuous variables with statistically significant results.

Predictor	N	Test/Model	Estimate/Effect	*p*-Value	Note
Self-reported travel costs (overall, €)	169	Logistic (univariable)	β = −0.053; OR = 0.948 (95% CI 0.907–0.991)	**0.025**	Higher costs → lower satisfaction
“Objective assessment” (yes vs. no)	285	Pearson χ^2^ (2 × 2)	χ^2^(1) = 6.999	**0.008**	Lower satisfaction is cited
“Lower costs” (yes vs. no)	285	Pearson χ^2^ (2 × 2)	χ^2^(1) = 6.671	**0.0098**	Lower satisfaction is cited

Notes: significant results at α = 0.05 are shown in bold; motives were analysed as separate binary items (multiple responses).

## Data Availability

The data that support the findings of this study are available from the corresponding author, M.M.H., upon reasonable request.

## Supplementary Materials

The following supporting information can be downloaded at: https://www.mdpi.com/article/10.3390/dj13110495/s1, Table S1: Univariable associations between candidate predictors and satisfaction (Yes vs No): χ^2^ tests for categorical variables and logistic regression for continuous variables with no statistically significant results.
