# Supplementary material for: Challenges of Future Patient Recruitment: A Cross-Sectional Study in Conservative Dentistry Teaching
Source: Dent J (Basel). 2025 Oct 25;13(11):495. doi: 10.3390/dj13110495 (PMC12651384; doi:10.3390/dj13110495)
Supplement: Supplementary file 1 [file dentistry-13-00495-s001.zip › dentistry-3865135-supplementary.pdf]

Supplement Table S1

Supplement Table S1: Univariable associations between candidate predictors and satisfaction (Yes vs No): χ^2 tests for categorical variables and logistic regression for continuous variables with no statistically significant results.

Predictor	N	Test/Model	Estimate / Effect	p-value	Note
Sex	285	Pearson χ^2 (2×2)	$\chi^2(1)=0.029$	0.866	n.s.
Age at participation (years)	285	Logistic	$\beta = -0.013$; OR ≈ 0.987	0.449	n.s.
One-way distance (km)	285	Logistic	$\beta = -0.0038$	0.678	n.s.
Per-appointment travel cost excl. parking (€)	285	Logistic	$\beta = -0.0073$	0.622	n.s.
Parking cost per appointment (€)	285	Logistic	$\beta = -0.0626$	0.582	n.s.
Discount / savings (“Minderung”, €)	285	Logistic	$\beta = -0.00168$	0.573	n.s.
Out-of-pocket contribution (€)	285	Logistic	$\beta = +0.00038$	0.889	n.s.
Net savings after travel+parking (€)	285	Logistic	$\beta = -0.00135$	0.644	n.s.
Mode of travel	285	Pearson χ^2	$\chi^2(4)=1.059$	0.901	Sparse cells
Motives for seeking students´ treatment: recommendation, proximity, knowledge level, no regular dentist, satisfaction with previous treatments, clinic notice, acquaintances, internet, insurer, leaflet, prior course participation	285	Pearson χ^2 (je 2×2)	—	all>0.05	n.s.
Questionnaire completed	285	—	No variability	—	All “Yes”
Notes: n.s. = not significant					
